# Psychosocial interventions for children with dermatological conditions: systematic review and meta-analysis

**DOI:** 10.1093/jpepsy/jsaf066

**Published:** 2025-08-31

**Authors:** Amy E Mitchell, Japheth O Adina, Alina Morawska, Emily Casey

**Affiliations:** School of Nursing, Midwifery and Social Work, The University of Queensland, St Lucia, QLD, Australia; Parenting and Family Support Centre, School of Psychology, The University of Queensland, St Lucia, QLD, Australia; Institute for Human Development, The Aga Khan University, Nairobi, Kenya; Parenting and Family Support Centre, School of Psychology, The University of Queensland, St Lucia, QLD, Australia; Dermatology Service, Children’s Health Queensland Hospital and Health Service, South Brisbane, QLD, Australia

**Keywords:** adherence/self-management, child, chronic illness, dermatology, health behavior, parenting, psychosocial intervention, quality of life, systematic review/meta-analysis

## Abstract

**Objective:**

Chronic skin conditions contribute to psychosocial difficulties and reduced child/parent quality of life, impacting condition management and disease control. The objective of this systematic review was to summarize the literature on psychosocial interventions (interventions that therapeutically target psychological/social processes to improve outcomes) for children with chronic dermatological conditions and their families.

**Methods:**

Searches of five electronic databases (CINAHL, PubMed, PsycINFO, Scopus, The Cochrane Library, Web of Science) identified relevant articles published from dates of inception to April 8, 2024, and reference lists were searched for additional relevant articles. Primary outcomes were disease/symptom severity and child quality of life. Interventions could be delivered in any format via controlled or uncontrolled studies. Articles had to report pre-post-intervention data and be published in English.

**Results:**

The review identified 10 eligible studies (reported in 12 papers) involving 2,346 families from seven countries. All reported on interventions for families of children with atopic dermatitis; none examined interventions for any other dermatological conditions. Eight studies evaluated face-to-face group-delivered interventions, and two studies evaluated self-directed online interventions. Meta-analyses revealed a significant effect on disease/symptom severity (standard mean difference = −0.34, 95% confidence interval = −0.53 to −0.15, *z* = 3.50, *p* < .001, *I*^2^ = 74%) but no significant effect on children’s quality of life (standard mean difference = −0.09, 95% confidence interval = −0.26 to 0.09, *z* = 0.99, *p* = .32, *I*^2^ = 42%). Effects on secondary (parent and family) and other outcomes were mixed.

**Conclusions:**

Psychosocial interventions may help to improve disease/symptom severity and other important outcomes for families of children with atopic dermatitis. Future research should examine efficacy in other pediatric dermatological conditions.

Chronic dermatological conditions are common in childhood and represent a significant disease burden globally (Karimkhani et al., 2017; Yakupu et al., 2023). Medical and other treatments continue to evolve; however, children and their families still experience difficulties with treatment access and day-to-day implementation (Ablett & Thompson, 2016; Prindaville et al., 2015; Toy et al., 2021), and medical treatments do not address the psychosocial problems which many children and parents experience (Carleen et al., 2022). Understanding the psychosocial challenges faced by children and their families and empowering them to take charge of their own wellbeing and emotional health is now seen as a top priority in pediatric dermatology research (Neale et al., 2023).

Dermatological conditions in childhood, such as atopic dermatitis (AD; synonymous with “atopic eczema”), psoriasis, and vitiligo, are associated with behavioral, emotional, and social problems, stigmatization, and reduced quality of life (QoL; Ablett & Thompson, 2016; Baker & Billick, 2022; Hu et al., 2020; Kelly et al., 2021; Kern et al., 2021; Mian et al., 2019; Olsen et al., 2016; Strouphauer et al., 2023). AD is the most common, affecting approximately 15%–20% of children and adolescents, with global estimates indicating median current prevalence of 6% and 1% for eczema and severe eczema, respectively (Langan et al., 2023). The etiology of AD is yet to be fully understood, but involves complex interactions between biological and environmental factors that contribute to skin barrier dysfunction, increased reactivity and inflammation, dryness, and intense pruritus (itch; Thyssen et al., 2020). Scratching damages the skin, increases risk of infection, and contributes to disrupted sleep for children and parents, appearance-related stigmatization and teasing, and impaired QoL (Capozza et al., 2020; Cortés et al., 2022; Kelly et al., 2021; Olsen et al., 2016).

As for many chronic dermatological conditions, clinical guidelines for pediatric AD recommend regular, consistent treatment that steps up or down as indicated. This includes daily emollient (moisturizer), even when skin is clear; addition of topical corticosteroids for mild AD; and a step-up to of calcineurin inhibitors, bandaging/wet-wrapping, and phototherapy and/or systemic therapy for moderate to severe disease (National Institute for Health Care Excellence, 2023). While consistency is key to successful management, treatment can be complex, costly, and time-consuming, and two-thirds of children do not adhere to their treatment plan (Ellis et al., 2011; Krejci-Manwaring et al., 2007). Discomfort (e.g., itching, stinging, burning) during treatment is distressing for children and parents alike, and child resistance (e.g., refusal, complaining, tantruming) is commonly problematic, contributing to inadequate or missed treatment (Santer et al., 2013; Teasdale et al., 2021). Psychosocial factors are highly relevant to the AD management context, as more severe AD is associated with greater internalizing and externalizing child behavior problems, increased parenting stress, more parenting difficulties, lower self-efficacy for managing the child’s condition, and less successful treatment implementation (Mitchell et al., 2015, 2016; Santer et al., 2014).

Psychosocial challenges experienced by children and families are often underestimated or unrecognized in dermatology practice, and there have been calls to improve understanding of mental health burden and ensure appropriate treatment or referrals to support patients and families (Connor, 2017; Cortés et al., 2022; De Vere Hunt et al., 2020; Mian et al., 2019; Mitchell, 2018; Strouphauer et al., 2023) including the development of psychosocial screening/assessment tools (Carleen et al., 2022; Russell et al., 2022). Likewise, the importance of considering children’s wellbeing beyond the immediate context of their medical treatment has been increasingly recognized, and development and testing of psychosocial interventions, including those to support parents, have been noted as a priority (Chatrath et al., 2023; Mitchell, 2018; Morawska et al., 2015; Neale et al., 2023). Given the importance of psychosocial health and wellbeing to patients and caregivers across the spectrum of dermatologic disease (Neale et al., 2023), there is a compelling rationale for taking a cross-diagnostic approach to understanding effective ways to support children and families.

To our knowledge there are no prior systematic reviews of psychosocial interventions for children with dermatological conditions, although reviews of psychosocial interventions in the context of other chronic pediatric conditions show promise in alleviating psychosocial burden and improving health outcomes (Fisher et al., 2014; Janicke et al., 2014; Law et al., 2014; Lohan et al., 2015; Mitchell et al., 2020). We need a clearer picture of the current state of the evidence for the efficacy of psychosocial interventions to guide policy and practice in the treatment of childhood dermatological conditions. This systematic review aimed to answer the question of how effective psychosocial interventions are in improving disease and symptom severity, treatment adherence, and psychosocial outcomes for children with chronic dermatological conditions and their families. For the purposes of this study, “psychosocial intervention” was defined as any intervention that incorporates a therapeutic intervention component that facilitates change by targeting psychological or social processes to improve child, parent, or family outcomes. These may include (but are not limited to) cognitive behavioral or behavior change interventions, counseling, parenting support, or peer support interventions.

## Methods

This review was conducted according to the Preferred Reporting Items for Systematic Reviews and Meta-Analyses (PRISMA) guideline (Moher et al., 2009) and prospectively registered with PROSPERO (2021 CRD42021226302). The final PRISMA checklist is available as Supplementary File S1.

### Search strategy

We performed initial searches between January and February 2021 and updated the search on 8 April 2024. Searches were conducted across five electronic databases (CINAHL, PubMed, PsycINFO, Scopus, The Cochrane Library, and Web of Science) using titles and abstracts to identify published articles relevant to our systematic review question. The search terms (see Table 1) were defined to match the requirements of each database. Although “dermatology*” and “skin” were used to capture a broad range of conditions, the most common pediatric chronic dermatological conditions were also included as separate terms to improve the sensitivity of the search. No restrictions or filters were put on our search strategy in terms of date and geographic location.

**Table 1.** Search terms.

[1] child* OR neonat* or infant* OR baby OR babies OR toddler* OR pre-school* OR preschool* OR school-age* OR youth* OR pre-adolescent* OR adolescent* OR teen* OR paediatric* OR pediatric* AND [2] intervent* OR program* OR support* OR training OR practice* OR skill* OR educat* OR therap* OR behaviour* OR behavior* OR cognitive OR support* OR psycho* OR manage* OR counsel* OR autogenic OR relaxation OR biofeedback OR ‘family therapy’ OR ‘health promotion’ OR self-help OR ‘self-help’ OR mindfulness OR imagery AND [3] dermatolog* OR skin OR eczema OR ‘atopic dermatitis’ OR neurodermatitis OR psoriasis OR vitiligo OR ‘chronic urticaria’ OR ‘alopecia areata’ OR ‘epidermolysis bullosa’ OR acne

### Eligibility criteria and study selection

Articles were imported into Endnote and screened for inclusion based on title and abstract by J.O.A. Full-texts of potentially relevant articles were retrieved and independently assessed against eligibility criteria by J.O.A. and A.E.M., with only one disagreement that was resolved through discussion among the authors. Inclusion and exclusion criteria were as follows:

#### Population and intervention

Psychosocial interventions that were delivered to the parents or family of a child (<18 years of age) with a chronic dermatological condition, or to the child themselves, were eligible for inclusion. Articles that reported on interventions with mixed child-adult samples were excluded unless separate data for the subsample of child participants were included.

#### Format

Articles reporting on interventions delivered via any format (e.g., individual, group, online self-directed) and any dosage (e.g., access to online modules, 10 group sessions) were eligible for inclusion.

#### Comparator

Study designs could include any comparison group (e.g., wait-list, care as usual, education) or no comparison group.

#### Type of studies

Randomized controlled trials (RCTs) or uncontrolled studies reporting pre- and post-intervention data, and published in English language, were eligible for inclusion. Articles missing pre- and/or post-intervention data, or not published in English, were excluded.

#### Outcomes

Primary outcomes were disease or symptom severity (objective or clinician-rated disease severity, or child self-report or parent-report of disease/symptom severity) and child QoL. Secondary outcomes were parenting skills, parenting efficacy, parent mental health, impact of condition, and family QoL, and may also include sibling outcomes (knowledge, behavioral and emotional adjustment, QoL, impact of the condition). Articles not reporting on at least one primary outcome were excluded.

### Data extraction and risk of bias assessment

We extracted data on study characteristics for each of the eligible articles, including study setting, study design, participant characteristics, intervention description, and outcomes. Data extraction and information coding were conducted independently by J.O.A. and A.E.M. The quality of each study (risk of bias) was assessed independently by J.O.A. and A.E.M. using the Cochrane Collaboration’s Risk of Bias (RoB) tool for randomized trials (Higgins et al., 2019), and disagreements were resolved through discussion with a third reviewer. The revised tool to assess risk of bias in randomized trials (RoB2) was used to generate graphs depicting risk of bias within and across studies (Sterne et al., 2019).

### Data analysis

We performed a meta-analysis using the Cochrane Collaboration’s Review Manager Version 5.4 (2020). The standard mean differences for continuous data were computed, and random-effects models were used to estimate the overall intervention effect sizes. We only meta-analyzed outcome data for disease/symptom severity (assessed using the SCORing Atopic Dermatitis [SCORAD] visual assessment tool or the Patient-Oriented Eczema Measure [POEM]) and child QoL (assessed using the Children’s Dermatology Life Quality Index [CDLQI] or Infants’ Dermatitis Quality of Life Index [IDQOL] questionnaires) since there was inconsistency in measurement and insufficient data for other study outcomes such as parental self-efficacy, parental mental health, and parent/family QoL, which are qualitatively summarized in this review.

Effect sizes are reported using standardized mean differences with 95% confidence intervals (CI) for continuous outcomes. We report random-effects inverse-variance-weighted mean effect sizes and 95% CIs using forest plots. Random-effects models account for variability in population parameters of studies and allow for the generalization of meta-analytic results (Field & Gillett, 2010; Hunter & Schmidt, 2000). Studies were only considered for meta-analysis when the outcome measures and assessment were comparable between the studies. Only one study used a measure other than SCORAD or POEM as the main indicator of disease/symptom severity (an unvalidated 0–10 parent rating of AD severity; Heapy et al., 2022) and was excluded from the meta-analysis. Where studies used SCORAD or POEM as the main indicator of disease/symptom severity but also employed a secondary parent-report measure, we noted whether there were reported improvements on these outcomes, but they were not included in the meta-analysis. The data for meta-analysis were drawn from data collected at the pre- and post-intervention assessment timepoints. We could not conduct a meta-analysis on longer-term follow-up outcome data since most of the studies did not collect or report on follow-up data beyond the post-intervention timepoint.

Statistical heterogeneity between studies was assessed using the *I^2^* statistic. The corresponding values of the *I^2^* statistic normally represent low heterogeneity if less than 25%, and substantial or high heterogeneity if 50% or more (Shuster, 2011). The Tau^2^ statistic was used to determine the amount of estimated variance of the true effect sizes of studies included in the meta-analyses. Since there were fewer than 10 eligible studies reporting group-level data, we could not use the funnel plot method to assess for publication bias. Finally, the Grading of Recommendations Assessment, Development, and Evaluation (GRADE) criteria were applied to evaluate the overall quality of the evidence. The data extracted from studies in this systematic review are freely available in the published peer-reviewed journal articles in which the studies are reported. All data analyzed during this study are included in this published article.

## Results

### Study selection

**Figure 1.**
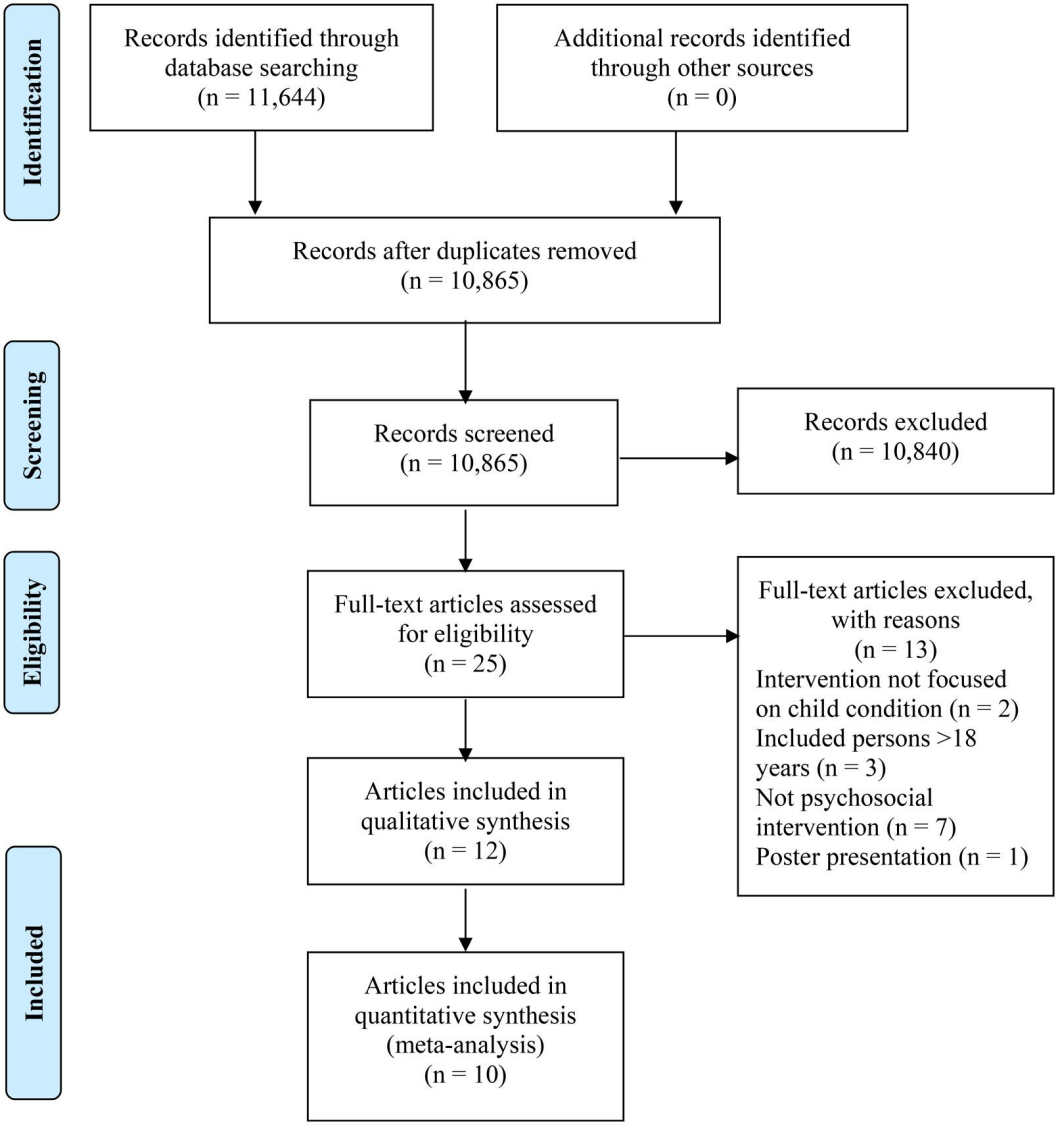
PRISMA diagram of studies included in the review.

The search yielded 11,644 records (see Figure 1). After removing duplicates, 10,890 references were screened by title and abstract and 10,865 were excluded. We reviewed 25 articles based on full-text and 13 were excluded (reasons presented in Figure 1). One was not a published article (Muller et al., 2021), two were not focused on the child’s condition (Weisshaar et al., 2008; Yoo et al., 2018), three included adult patients without disaggregated child data (Armstrong et al., 2015; Balato et al., 2013; Mollerup et al., 2014) and seven were not evaluations of a psychosocial intervention (Ersser et al., 2013; Futamura et al., 2013; Grillo et al., 2006; Koch et al., 2008; Koutroulis et al., 2014; LeBovidge et al., 2021; Thomas et al., 2017).

The remaining 12 articles reporting on 10 separate studies met the eligibility criteria and were included in this review. All included studies were RCTs except one, which used a single-case experimental design (Heapy et al., 2022). The 10 studies were from seven countries, with three (reported in four papers) from Germany, two from China, two from UK, one (reported in two papers) from Australia, one from Brazil and one from Croatia.

### Participant characteristics

Table 2 presents the details of study characteristics. Overall, the review included 2,346 participants from 10 separate studies and the samples ranged from 7 to 823 families. The children involved in the studies were aged 3 months to 18 years, with most samples comprising more boys than girls. Of the six studies that reported parent age or sex, parents were aged 20–50 years, and 86%–94% of primary participants were mothers. Only five studies reported any indicator socioeconomic status: most parent participants had a tertiary qualification (40%–100% of each sample; Brunner et al., 2023; Heapy et al., 2022; Morawska et al., 2016, 2017; Santer et al., 2022; Xie et al., 2020) and Morawska et al. (2016) reported that 83% of families were able to meet their essential expenses over the past 12 months. Ethnicity was reported in only three studies (two from the UK, one from Australia), with each sample comprising >80% Caucasian/White participants. Gender identity and disability status were not reported in any studies.

**Table 2.** Study characteristics.

Authors, country, study design	Participant characteristics	Intervention details	Assessment, outcomes, and measures	Reported results
Brunner et al. (2023) Germany RCT	Parents of children with AD, recruited via hospital clinic *N *= 40 Intervention *n *= 21 Control *n *= 19 Parent age: 26–44 years (90% mothers) Child age: 0–5 years (55% boys)	*Name:* Not reported *Delivered to:* Parents only *Description:* Self-directed online video-based education program using storytelling Six videos (each 3–6 min) created by multi-professional team. Includes storytelling (parents of children with AD sharing their experiences) and evidence-based information about AD (from specialist nurse, pediatric dermatologist, psychologist) Content includes causes of AD symptoms, skincare and treatment instructions, demonstration of a treatment plan, and information about health and emotional consequences of uncontrolled AD and itching Followed by expert consultation (pediatric dermatologist and/or advanced practice nurse) after post-intervention assessment	Assessment at pre- and post-intervention, 3-month follow-up *Parent/family outcomes:* Parent fear of topical corticosteroids (TOPICOP) Parent QoL (FDLQI) *Child outcomes:* AD severity (SCORAD)	*Parent/family outcomes:* Reduction in parent fear of topical corticosteroids (Worries, Beliefs, and Total scores) for intervention group at post-intervention, maintained to 3-month follow-up No effect on parent QoL *Child outcomes:* No effect on AD severity
Heapy et al. (2022) UK Single-case experimental design	Parents of children with AD (*n *= 6) or psoriasis (*n *= 1) *N *= 7 Parent age: 30–50 years (86% mothers) Child age: 4–12 years (57% girls) Ethnicity: White British: 100%	*Name:* Mindful Parenting *Delivered to:* Parents only *Description:* Group-delivered mindful parenting intervention 8 × 3-h group sessions, delivered over 10 weeks (university clinic setting) Based on mindfulness-based cognitive therapy and mindful-based stress reduction program. Provides training in applying mindfulness skills to the task of parenting Session structure: review of the home practice, formal meditation practice (e.g., a body-scan meditation), mindful parenting exercise (e.g., observing your child with beginner’s mind), group discussion around a theme	Assessment at pre- and post- intervention, 6-week follow-up *Parent/family outcomes:* Parenting stress (idiographic, 0–100 scale) Parent stress (PSI-SF-4) Parent depression (PHQ-9) Parent anxiety (GAD-7) Parent QoL (FDLQI) *Child outcomes:* Parent-rated AD severity (0–10 scale) Child-reported QoL (CDLQI)	From pre-intervention to 6-week follow-up: *Parent/family outcomes:* Improved parenting stress Stress improved for 3/7 parents Depression improved for 1/7 parents Anxiety improved for 3/7 parents QoL improved for 1/7 parents *Child outcomes:* AD severity reduced for 4/7 children QoL improved for 1/7 children
Kupfer et al. (2010) Germany RCT	Children with atopic dermatitis (AD) and their parents *N *= 185 Intervention *n *= 102 Control *n *= 83 Child age: 8–12 years (56% boys) Parent age, gender, and ethnicity not reported	*Name:* Not reported *Delivered to:* Parents and children (parallel sessions) *Description:* As for Staab et al. (2006)	Assessment at pre- and post-intervention, 12-month follow-up *Parent/family outcomes:* Parenting behavior (FEN) *Child outcomes:* Child-reported: Coping (COPEKI) Itching-scratching cognitions (JUCKKI)	From pre-intervention to 12-month follow-up: *Parent/family outcomes:* Improvements in parenting behavior (aggression against scratching, control of scratching, negative treatment experience) but not in overprotective behavior *Child outcomes:* Improvements in coping (social anxiety/depressive mood, itching-scratching circle/stress from the disease) Improvements in itching-scratching cognitions (catastrophizing, coping)
Liang et al. (2018) China RCT	Children with moderate to severe AD (SCORAD > 20) and their parents *N *= 542 Intervention *n *= 293 Control *n *= 249 Child age: 2–14 years (54% boys) Parent age, gender, and ethnicity not reported	*Name:* Not reported *Delivered to:* Parents and children *Description:* Therapeutic patient education program 4 × 2-h weekly group sessions (30–40 participants per group) Delivered by a multidisciplinary team (hospital setting) Session structure: long-term treatment and management of AD, food allergy and AD, how to increase the family happiness index of patients using psychological interventions, skin care, use of emollients for AD Delivered by pediatric dermatologists, psychologist, advanced practice nurse Opportunity for participants to consult and discuss with instructors after the session Families provided with video recording of lecture and printed materials to take home, and provided with therapy/diet recommendations based on severity	Assessment at baseline, 3- and 6-month post-intervention *Parent/family outcomes:* Knowledge of emollients (questionnaire designed for study) *Child outcomes:* AD severity (SCORAD) QoL (IDQOL/CDLQI)	*Parent/family outcomes:* Improved knowledge of emollients from baseline to 6 months post-intervention *Child outcomes:* AD severity improved at 3- and 6-month post-intervention QoL improved at 3- and 6-month post-intervention for children aged 2–4 years but not 5–14 years
Morawska et al. (2016) Australia RCT	Parents of children with asthma and/or AD AD subsample *N *= 85 Intervention *n *= 39 Care as usual *n *= 46 Parent age: 24–48 years (94% mothers) Child age: 2–10 years (52% boys) Ethnicity Caucasian: 86% Asian: 9% Pacific islander: 1% Not specified: 4%	*Name:* Positive Parenting for Healthy Living *Delivered to:* Parents only *Description:* Parenting intervention 2 × 2-h weekly discussion group sessions Delivered by psychologist or nurse (university setting) Draws on the theoretical principles that form the basis of the Triple P-Positive Parenting Program and developed for families of children with chronic health conditions Uses didactic instruction, video modeling, active skills training, and homework tasks Session 1: Strategies to prevent and manage problem behaviors, ensure illness prevention and management plans implemented appropriately: continuing regular activities, having realistic expectations, reducing stress, helping siblings cope, condition-specific management steps, involving the child, communicating with caregivers, keeping track of symptoms, being prepared for emergencies Session 2: Principles of positive parenting: promote positive parenting practices, develop effective disciplinary methods, create environments conducive to caring parent–child relationships—causes of behavior problems in children with chronic illnesses, parenting traps, use of routines to improve treatment management, preventing and managing anxiety and behavioral strategies (descriptive praise, positive attention, clear instructions, consequences, behavior charts) Take-home tip sheet(s) provided information on disease etiology and management	Assessment at pre- and post-intervention, 6-month follow-up *Parent/family outcomes:* Self-efficacy with AD management Self-efficacy with AD behavior management Competence with AD management (observed) Parent HRQoL Family HRQoL *Child outcomes:* AD behavior difficulties (EBC Extent) AD symptom severity (POEM) HRQoL (PedsQL)	From pre-intervention to 6-month follow-up: *Parent/family outcomes:* Improved self-efficacy with AD management Improved self-efficacy with AD behavior management No effect on competence with AD management Improved parent HRQoL Improved family HRQoL *Child outcomes:* Reduced AD behavior difficulties Reduced AD symptom severity No effect on HRQoL
Morawska et al. (2017) Australia RCT	Fathers of children with asthma and/or AD AD subsample *N *= 47 Intervention *n *= 24 Care as usual *n *= 23 Parent age: 30–44 years (100% fathers) Child age: 2–10 years (63% boys) Ethnicity: Caucasian: 91% Asian: 5% African: 2% Not specified: 2%	*Name:* Positive Parenting for Healthy Living *Delivered to:* Parents only *Description:* as for Morawska et al. (2016) Secondary data from fathers who participated alongside mothers (the primary participants; Morawska et al., 2016)	Assessment at pre- and post-intervention, 6-month follow-up *Parent/family outcomes:* Self-efficacy with AD management Self-efficacy with AD behavior management Parent HRQoL Family HRQoL *Child outcomes:* AD behavior difficulties (EBC Extent) HRQoL	From pre-intervention to 6-month follow-up: *Parent/family outcomes:* Improved self-efficacy with AD management No effect on self-efficacy with AD behavior management No effect on parent HRQoL No effect on family HRQoL *Child outcomes:* Reduced AD behavior difficulties Improved HRQoL
Pustišek et al. (2016) Croatia RCT	Parents of children with moderate or severe AD (SCORAD > 25) *N *= 158 Intervention *n *= 64 Control *n *= 64 Parent age: 20–42 years (93% mothers) Child age: 3 months–7 years (59% boys)	*Name:* Not reported *Delivered to:* Parents only *Description:* Brief structured educational program Single group session comprising 2-h lecture and written material (5–8 participants per group) Delivered by physician specialist in dermatology (hospital setting); team that developed the lecture included psychologist and clinical dietician Causes of atopic disease, allergic and non-allergic factors leading to deterioration of clinical course, recommended nutrition, required diagnostic tests, correct skin care and treatment, impact of disease on QoL of child and family, relaxation methods to improve sleep and alleviate pruritic Followed by nurse presentation on correct use of topical treatment, moist bandages, and skin care Supplemented with educational booklet and diary to record corticosteroid use	Assessment at pre-intervention and 2-month follow-up *Parent/family outcomes:* Stress (PSS) Anxiety (STAI) Parent QoL (FDLQI) *Child outcomes:* AD severity (SCORAD) Parent-rated AD severity (patient-oriented SCORAD)	By 2-month follow-up: *Parent/family outcomes:* Reduced stress Reduced state anxiety but not trait anxiety Reduced impact on parent QoL overall *Child outcomes:* Improved physician-assessed AD severity Improved parent-rated AD severity, reduced pruritis and sleep disturbance
Santer et al. (2022) UK RCT	Parents of children with mild to severe eczema (POEM > 5) *N *= 340 Intervention *n *= 171 Usual care *n *= 169 Parent age: no range reported, *M *= 38 years (92% mothers) Child age: 0–12 years (52% boys) Ethnicity: White: 83% Asian: 7% Black: 3% Mixed: 4% Other: 2% Not reported: 1%	*Name:* Eczema Care Online *Delivered to:* Parents only *Description:* Self-guided online behavioral intervention to support AD self-management Aims to reduce eczema severity and target core behaviors: emollient and topical corticosteroid use, avoidance of irritants and triggers, minimization of scratching, emotional management Participants complete core section comprising key information and behavior change content before accessing main menu with various topics of interest Tailored content delivery via suggestion of topics of relevance, includes interactive and audiovisual features (e.g., brief eczema assessment, videos, stories, advice from families of children with AD) Co-produced by a team of behavioral psychologists, patient representatives, general practitioners, dermatology nurse consultants, dermatologists	Assessment at pre-intervention, 24- and 52-week follow-up. *Parent/family outcomes:* Enablement (Patient Enablement Instrument) Barriers to treatment use (Problematic Experiences of Therapy Scale) Treatment adherence (self-report) *Child outcomes:* Parent-rated AD symptom severity (POEM) Eczema control (RECAP) QoL (CHU-9D)	*Parent/family outcomes:* Enablement improved at 24 weeks, sustained to 52 weeks No effects at 24 weeks; reduced doubts about treatment efficacy (only) at 52 weeks No effects at 24 weeks; improved use of daily emollient (only) at 52 weeks *Child outcomes:* AD symptom severity improved over 24 weeks, sustained to 52 weeks No effect on eczema control No effect on QoL
Staab et al. (2002) Germany RCT	Parents of children with moderate to severe AD (SCORAD > 20) *N *= 204 Intervention *n *= 93 Control *n *= 111 Child age: 5 months–12 years Parent age, parent sex, child sex, and ethnicity not reported	*Name:* Not reported *Delivered to:* Parents only *Description:* Structured parental training program 6 × 2-h weekly group sessions Covered medical, nutritional, and psychological topics with the aim of transferring the knowledge to daily life at home; participants encouraged to share experiences and practice new skills Delivered by interdisciplinary team of pediatricians, psychologists, nutritionists Session 1: Introduction, medical information about AD, introduction of a relaxation technique Session 2: Recognition and avoidance of triggers, daily skin care Session 3: Stress management, dealing with itching and scratching and sleep disturbances Session 4: Stage related treatment of symptoms, unconventional therapies Session 5: General child nutrition, food allergies in AD, different forms of diets Session 6: Issues of coping, self-management plan, problems in transfer to daily routine	Assessment at pre-intervention and 1-year follow-up *Parent/family outcomes:* Treatment use and condition management General and AD-specific QoL (Daily Life, new AD-specific QoL questionnaire) Coping strategies (Trier Scales of Coping) *Child outcomes:* AD severity (SCORAD)	*Parent/family outcomes:* Greater use of regular skin care, antiseptics, topical corticosteroids; reduced dietary restriction in absence of proven allergy; increased allergen avoidance No effect on general or AD-specific QoL No effect on coping strategies *Child outcomes:* No effect on AD severity
Staab et al. (2006) Germany RCT	Children with AD and their parents Overall *N *= 827 Intervention *n *= 446 Control *n *= 377 3 months–7 years: Intervention *n *= 274 Control *n *= 244 (52% boys) 8–12 years: Intervention *n *= 102 Control *n *= 83 (56% girls) 13–18 years: Intervention *n *= 70 Control *n *= 50 (61% girls) Parent age, parent sex, and ethnicity not reported	*Name:* Not reported *Delivered to:* For children 3 months–7 years, intervention delivered to parents only For children 8–12 years, parallel sessions for parents and children For adolescents 13–18 years, intervention delivered to adolescents only (parent attendance optional for sessions 1 and 2) *Description:* as for Staab et al. (2002)	Assessment timepoint: Pre-intervention and 12-month follow-up *Parent/family outcomes:* QoL (Quality of Life in Parents of Children with Atopic Dermatitis; assessed for parents of children 3 months–12 years only) *Child outcomes:* AD severity (SCORAD) Parent-rated AD severity (Skin Detective) Child-reported itching-scratching cognitions (JUCKKI/JUCKJU; assessed for children 8–12 years and 13–18 years only)	*Parent/family outcomes:* Confidence in medical treatment, emotional coping, and acceptance of disease improved for parents of children 3 months–7 years and 8–12 years; psychosomatic wellbeing and effects on social life improved for parents of children 3 months–7 years only *Child outcomes:* AD severity improved (all age groups) Parent-rated AD severity improved (all age groups) Itching-scratching catastrophization improved for children 8–12 and 13–18 years; coping improved for children 8–12 years only
Weber et al. (2008) Brazil RCT	Children with moderate to severe AD *N *= 32 Intervention *n *= 16 Control *n *= 16 Child age: 2–16 years (56% male) Parent age, parent sex, and ethnicity not reported	Name: *Not reported* *Description:* AD support groups *Delivered to:* Parents and children (parallel sessions) Parents and children attended 90-min support group meetings every 2 weeks for 6 months Parent meetings: Coordinated by dermatologists. Started with explanation of the subject selected for the session, with a writing text to stimulate discussion, sharing of experiences, guided discussion Child meetings: Coordinated by child psychiatrist and volunteer medical students. Started with 30-min free play, followed by brief education on disease and treatment, discussion on a theme, and play activity related to the subject (playing, drawing, simulations, performances)	Assessment at pre- and post-intervention *Parent/family outcomes:* Family QoL (FDI) *Child outcomes:* Pruritus QoL (CDLQI)	*Parent/family outcomes:* No effect on QoL *Child outcomes:* Reduced frequency of pruritis, reduced effect of pruritis on mood Improved QoL
Xie et al. (2020) China RCT	Children with AD and their parents *N *= 113 Intervention *n *= 58 Control *n *= 55 Parent age: range not reported (*M *= 41 years) (88% mothers) Child age: 6–12 years (53% boys) Ethnicity not reported	*Name:* Not reported *Description:* Integrative body-mind-spirit (IBMS*)* group intervention *Delivered to:* Parents and children (parallel sessions) 6 × 3-h weekly group sessions, parallel sessions for parents and children (8–10 per group) Based on a holistic perspective and Eastern philosophies on health and wellbeing; emphasizes treating the whole person, balance between physical health, emotion, spirituality, and wider environment Delivered by social workers (social service center settings) Parent sessions: (1) Fundamentals of IBMS in AD; (2) Relationship between health and emotion; (3) Empower coping flexibility through experience sharing; (4) Discrepancies between parents’ and children’s experiences of coping with AD; (5) Reconstruct meaning of caregiver experiences; (6) Discover reciprocity and appreciation in the caregiver process Child sessions: (1) Explore self-identify, self-appreciation; (2) Disease-identity; (3) Improve recognition, expression, and regulation of emotion; (4) Enhance resilience capacity; (5) Identify personal strengths and resources; (6) Support networks Included 30-min period for joint parent–child activities (mindful jar making, problem-solving games, gift presentation, appreciation dialogue)	Assessment at pre- and post-intervention, 5-week follow-up *Child outcomes:* AD severity (SCORAD) Child-reported: Generalized and social anxiety symptoms (SCAS) Emotion regulation (ERC) Self-esteem (RSES) Quality of relationships with parents (CPS) QoL (CDLQI)	*Child outcomes:* Decreased AD severity at post-intervention but not sustained to 5-week follow-up Decreased generalized and social anxiety symptoms at 5-week follow-up only Improved lability/negativity at post-intervention and 5-week follow-up, improved emotion regulation at 5-week follow-up only No effect on self-esteem No effect on quality of relationships with parents No effect on QoL

All studies reported on interventions for children with AD. One study included a mixed sample of children with AD, asthma, or both conditions (Morawska et al., 2016, 2017) and another (Heapy et al., 2022) also included a single child with psoriasis. No studies examined interventions for any other dermatological conditions. Half of the studies (*k *= 5) restricted their samples to families of children with at least mild or moderate to severe AD. AD disease and symptom severity were assessed using a variety of validated measures. Six studies used SCORAD to assess clinician-rated disease severity (Brunner et al., 2023; Liang et al., 2018; Pustišek et al., 2016; Staab et al., 2002, 2006; Xie et al., 2020). Two of these (Pustišek et al., 2016; Staab et al., 2006) used additional measures to assess parent-rated disease severity (patient-oriented SCORAD and Skin Detective, respectively). Two studies used the Patient-Oriented Eczema Measure (POEM) to assess parent-reported symptom severity (Morawska et al., 2016; Santer et al., 2022), and one of these (Santer et al., 2022) used an additional measure (RECAP) to assess parent-reported AD control. One study (Heapy et al., 2022) used an unvalidated 0–10 scale to assess parent-rated disease severity, and another study (Weber et al., 2008) used a 0–10 visual analog scale to assess severity of pruritus.

### Intervention characteristics

The studies reported on nine different interventions: seven face-to-face group-delivered interventions and two self-directed online interventions. All had discrete psychosocial intervention components, most commonly teaching and practice of relaxation and stress management techniques, coping strategies, and emotion management; mindfulness training; evidence-based parenting support; or learning from other parents’ experiences via videos or group discussion. A detailed overview of each intervention is provided in Table 2. Overall, the interventions sought to support families to achieve good AD management, reduce AD severity, improve child and/or parent QoL, and improve parenting self-efficacy and mental health.

Most interventions (*k *= 6) were delivered to parents only. Two were delivered via parallel sessions for parents and children (Weber et al., 2008; Xie et al., 2020). One intervention was initially delivered to parents only (Staab et al., 2002) but later expanded to include parallel sessions for children 8–12 years and child-only delivery (with optional parent attendance at the introductory sessions) for adolescents aged 13–18 (Kupfer et al., 2010; Staab et al., 2006). No other interventions were delivered to children only.

#### Group interventions

The seven face-to-face group-delivered interventions were delivered in hospital (*k *= 3), university (*k *= 2), or social service center (*k *= 2) settings. They comprised between one and 12 sessions (median = 6 sessions), with each session lasting between 90 min and 3 h (median = 2 h) and total intervention time between 2 and 24 h (median = 12 h). Four interventions (reported in six papers) combined AD education with psychosocial intervention components (Kupfer et al., 2010; Liang et al., 2018; Pustišek et al., 2016; Staab et al., 2002, 2006; Weber et al., 2008) while three interventions (Heapy et al., 2022; Morawska et al., 2016, 2017; Xie et al., 2020) were predominantly psychosocial interventions. Interventions were delivered using diverse approaches, for example structured educational sessions, group discussion sessions, lectures and written materials, and psychotherapy sessions. Interventionists were from different professional backgrounds including psychology, social work, dietetics, nursing, and medicine (dermatology and psychiatry).

Four of the face-to-face group interventions combined evidence-based AD education with psychosocial intervention components. The studies conducted in Germany (2 studies reported in 3 papers; Kupfer et al., 2010; Staab et al., 2002, 2006) tested a structured training program that targeted key medical, nutritional, and psychological aspects of AD management (with parallel groups added for children 8–12 years and child-only delivery for adolescents aged 13–18). A separate study conducted in China (Liang et al., 2018) likewise tested a patient education program that combined AD education with psychosocial intervention. The briefest intervention, conducted in Croatia (Pustišek et al., 2016), tested a single 2-h lecture incorporating AD education with psychoeducation and relaxation skills training. The lengthiest intervention, conducted in Brazil (Weber et al., 2008), evaluated the effects of fortnightly parent and child support group meetings that combined AD education with group discussion on topics relevant to families of children with AD.

The remaining three face-to-face group-delivered interventions were predominantly psychosocial interventions. The study conducted in Australia and one of the studies conducted in the UK tested stand-alone parenting interventions: *Positive Parenting for Healthy Living* (based on the Triple P-Positive Parenting Program; Morawska et al., 2016, 2017), and *Mindful Parenting* (Heapy et al., 2022), which focused on supporting parenting and parent mental health. A separate study conducted in China (Xie et al., 2020) tested a stand-alone integrative mind-body-spirit group intervention delivered to parents and children in parallel groups.

#### Online interventions

The two online interventions were self-directed and delivered fully online. A study conducted in Germany (Brunner et al., 2023) tested a video-based education program that combined storytelling (parents of children with AD sharing their experiences) and AD education. A study conducted in the UK tested *Eczema Care Online* (Santer et al., 2022), a behavioral intervention that combined AD education with self-management and behavior change strategies that included videos, stories and advice from families of children with AD.

### Outcome measures

#### Child outcomes

Disease/symptom severity and child QoL were the primary outcomes for this review. Nine studies evaluated the effect of the interventions on disease/symptom severity (Brunner et al., 2023; Heapy et al., 2022; Liang et al., 2018; Morawska et al., 2016; Pustišek et al., 2016; Santer et al., 2022; Staab et al., 2002, 2006; Xie et al., 2020), most commonly via a clinician-rated visual assessment measure for disease severity (SCORAD) or a parent-reported questionnaire measure for symptom severity (POEM). Both are well-validated and widely used in AD research and clinical practice. Six studies evaluated the effect on child QoL, four via parent proxy-report (Liang et al., 2018; Morawska et al., 2016, 2017; Santer et al., 2022; Weber et al., 2008) and two via child self-report (Heapy et al., 2022; Xie et al., 2020).

Other child outcomes were also reported: eczema control, itching-scratching cognitions, pruritus, sleep disturbance, AD behavior difficulties, generalized and social anxiety symptoms, emotion regulation, self-esteem, coping, and quality of relationship with parents. Most were assessed via parent-report except for coping and itching-scratching cognitions (Kupfer et al., 2010; Staab et al., 2006) and anxiety, emotion regulation, self-esteem, and quality of the child-parent relationship (Xie et al., 2020), which were assessed via child self-report.

#### Parent and family outcomes

Parenting skills, parenting efficacy, mental health, impact of condition, and family QoL were the secondary outcomes for this review. One study examined effects on parenting behavior (Kupfer et al., 2010) while another examined effects on parent self-efficacy with AD management and AD-related child behavior management (Morawska et al., 2016, 2017). Two studies examined parental mental health (Heapy et al., 2022; Pustišek et al., 2016) and seven studies examined parent/family QoL (Brunner et al., 2023; Heapy et al., 2022; Morawska et al., 2016, 2017; Pustišek et al., 2016; Staab et al., 2002, 2006; Weber et al., 2008). Other parent outcomes were also reported: enablement, coping strategies, treatment adherence/condition management, knowledge of emollients, fear of topical corticosteroids, and barriers to treatment use.

### Risk of bias and quality of evidence assessments

**Figure 2.**
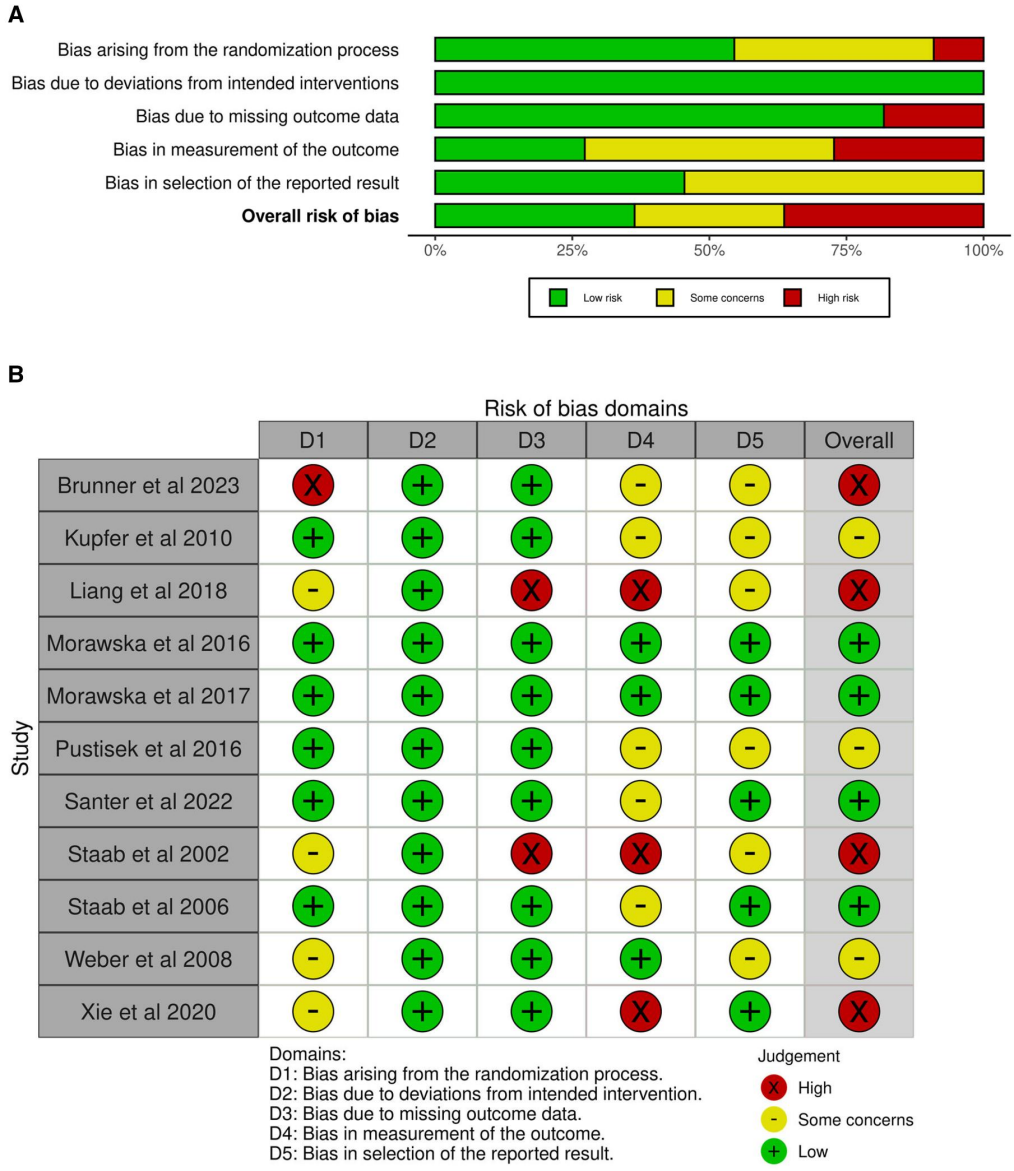
Risk of bias assessments (A) summarized by domain and (B) individual traffic plots.

Figure 2 summarizes risk of bias assessments for the RCTs. All had low risk of bias due to deviations from intended interventions, but some moderate/high risk of bias was evident across other domains. Overall, four (36.4%) studies had a high risk of bias, predominantly due to measurement of outcome (lack of blinding of disease severity outcome assessors, or lack of information on blinding; Brunner et al., 2023; Liang et al., 2018; Staab et al., 2002; Xie et al., 2020).

Table 3 summarizes results from the GRADE assessment. As per the GRADE approach, RCTs started as high-quality evidence and were primarily rated down due to imprecision of results (small sample sizes), risk of bias related to lack of blinding for disease severity outcomes, and heterogeneity in study results (for disease/symptom severity and parent QoL).

**Table 3.** Summary of findings from N = 10 studies assessed according to GRADE criteria.

Outcomes	Effects	No. of participants (studies)		Quality of the evidence (GRADE)	Notes
		Intervention	Comparison		
Disease/symptom severity	6 studies reported improvements in disease/symptom severity; 2 studies reported no change	1,156 (8 studies)	1,047 (8 studies)	⊕⊕⊖⊖^a^^,^^b^	
Child QoL	1 study reported improvement from pre- to post-intervention; 5 studies reported no change	546 (6 studies)	502 (5 studies)	⊕⊕⊕⊖^b^	
Parenting behavior (parent-report)	1 study reported improvement from pre- to 12-month follow-up	102 (1 study)	83 (1 study)	⊕⊕⊕⊖^c^	Improvements in aggression against scratching, control of scratching, and negative treatment experience, but not in overprotective behavior.
Parent self-efficacy	1 study reported improvements from pre- to post-intervention	39 (1 study)	46 (1 study)	⊕⊕⊕⊖^c^	Improved self-efficacy for managing AD for mothers and fathers. Improved self-efficacy for AD behavior management for mothers only.
Parent QoL	2 studies reported improvements from pre- to post-intervention; 1 study reported improvements for mothers but not fathers; 3 studies reported no change	600 (6 studies)	567 (6 studies)	⊕⊕⊕⊖^a^	
Parent mental health	2 studies reported improvements from pre- to post-intervention	71 (2 studies)	64 (1 study)	⊕⊕⊖⊖^b^^,^^c^	Over half of parents had reduced stress, parenting stress, and anxiety (but not depression) in 1 uncontrolled study; improved stress and state anxiety (but not trait anxiety) in 1 RCT.
Family QoL	1 study reported improvements from pre- to post-intervention for mothers but not fathers; 1 study reported no change	55 (2 studies)	62 (2 studies)	⊕⊕⊕⊖^c^	

*Note. AD* = atopic dermatitis; *GRADE* = Grading of Recommendations Assessment, Development, and Evaluation; *QoL* = quality of life; *RCT* = randomized controlled trial. GRADE Working Group grades of evidence: High quality ⊕⊕⊕⊕: Very confident that the true effect lies close to that of the estimate of the effect. Moderate quality ⊕⊕⊕⊖: The true effect is likely to be close to the estimate of the effect, but there is a possibility that it is substantially different. Low quality ⊕⊕⊖⊖: The true effect may be substantially different from the estimate of the effect. Very low quality ⊕⊖⊖⊖: The true effect is likely to be substantially different from the estimate of effect.

a Inconsistency (heterogeneity in study results).

b Limitations due to study design and implementation and associated risks of bias.

c Imprecision of results due to small sample sizes.

### Intervention effects

Meta-analysis compared data from participants allocated to intervention and control conditions at post-intervention. Post-intervention disease/symptom severity and child QoL mean scores and standard deviations (*SD*) together with the corresponding sample sizes (*n*) were extracted for the intervention and control arms of each study and are summarized in Table 4.

**Table 4.** Means and standard deviations for primary outcomes (disease/symptom severity and child quality of life scores) at post-intervention and summary of intervention effects.

Study		Intervention			Comparison			
	Disease severity	*n*	*M*	*SD*	*n*	*M*	*SD*	Effect (Y/N)
Brunner et al. (2023)	SCORAD	21	21.71	20.52	19	17.07	15.25	N
Liang et al. (2018)	SCORAD	293	20.71	15.37	249	22.94	15.67	Y
Pustišek et al. (2016)	SCORAD	64	23.08	15.19	64	36.44	16.76	Y
Staab et al. (2002)	SCORAD	72	24.00	15.92	73	26	16.82	N
Staab et al. (2006)	SCORAD	446	24.30	16.30	377	32.10	16.60	Y
Xie et al. (2020)	SCORAD	58	44.05	20.29	55	56.84	17.70	Y
	Symptom severity							
Morawska et al. (2016)	POEM	31	10.97	6.21	41	13.52	5.99	Y
Santer et al. (2022)	POEM	171	8.90	6.70	169	10.00	6.60	Y
	Child QoL							
Heapy et al. (2022) ^a^ ^,^ ^b^	CDLQI	7	–	–				N
Liang et al. (2018) ^c^	CDLQI	96	6.11	4.37	83	6.22	4.96	N
	IDQOL	178	5.53	4.19	151	6.09	3.92	N
Morawska et al. (2016)	PedsQL	44	73.27	14.94	51	74.84	14.99	N
Morawska et al. (2017)	PedsQL	31	77.6	12.75	24	77.41	14.37	N
Santer et al. (2022)	CHU-9D	116	0.90	0.09	122	.88	.10	N
Weber et al. (2008)	CDLQI	16	3.63	3.30	16	6.19	3.54	Y
Xie et al. (2020) ^a^	CDLQI	58	4.55	3.79	55	5.95	4.46	N

*Note*. *CDLQI* = Children’s Dermatology Life Quality Index; *CHU-9D* = Health-related quality of life; *IDLQI* = Infants’ Dermatitis Quality of Life Index; *N* = no—no improvement at post-intervention relative to comparison group (where applicable); *PedsQL* = Paediatric Quality of Life Generic Core Scale; *POEM* = Patient-Oriented Eczema Measure; *QoL* = quality of life; *SCORAD* = Scoring Atopic Dermatitis; *Y* = yes—improvement at post-intervention relative to comparison group (where applicable).

a Child self-report.

b No group-level data reported (case series, no comparison group).

c CDLQI for children aged 5–14 years, IDQOL for children aged 2–4 years.

**Figure 3.**
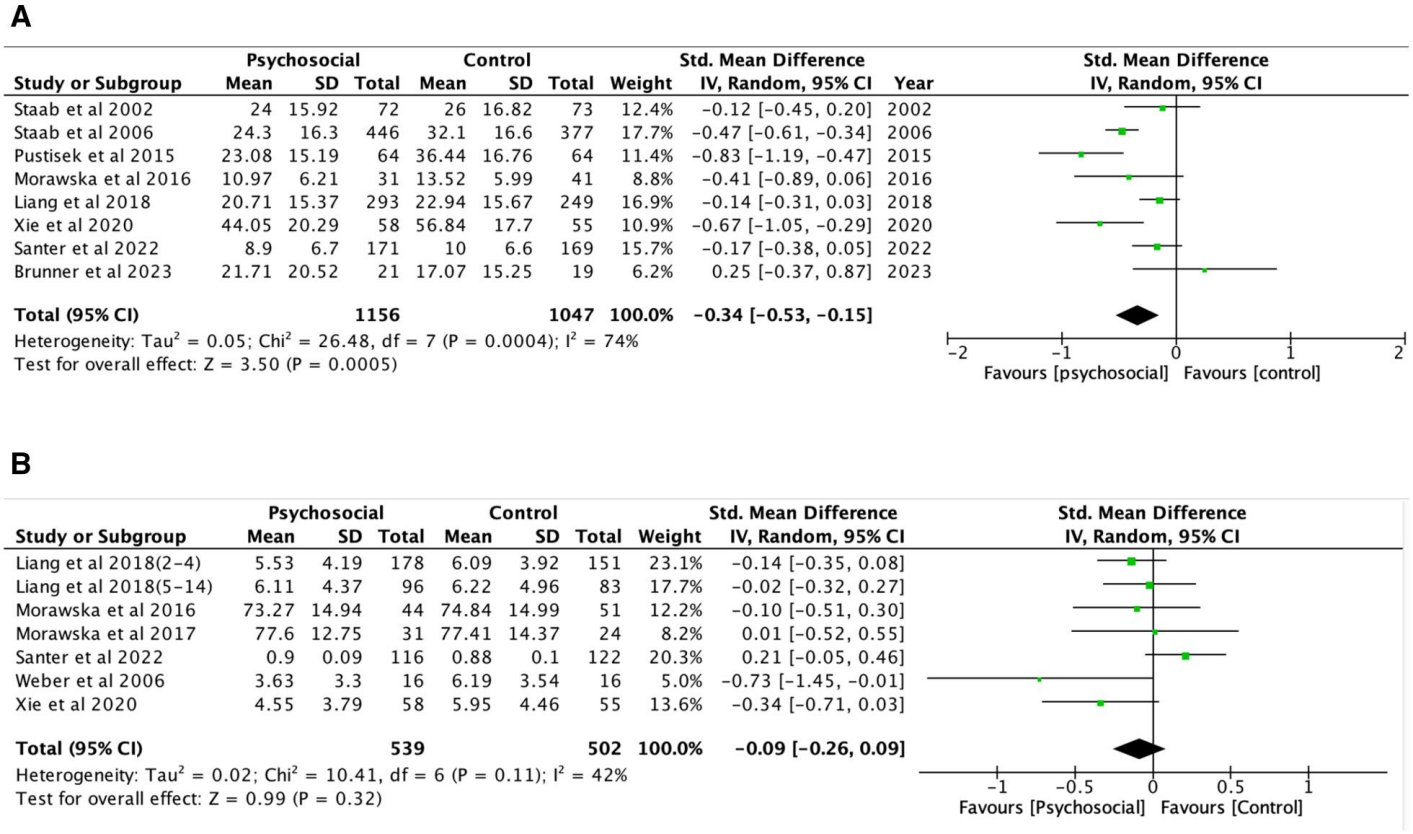
Meta-analytic results of psychosocial interventions for (A) disease/symptom severity and (B) child quality of life.

Meta-analysis of disease/symptom severity (Figure 3A) showed a statistically significant improvement from baseline to post-intervention (SMD = −0.34, 95% CI = −0.53 to −0.15; *z* = 3.50, *p* < .001; *I*^2^ = 74%). There was a high degree of heterogeneity (*I^2^* = 74%), which could be due to variability in measures and the small number of studies. The corresponding 95% CI was somewhat stable and ranged from a small to medium effect, thus yielding more confidence in the interpretation of the estimated treatment effect of the eight intervention studies included in the analysis. Three out of the eight studies showed statistically significant intervention effects on disease/symptom severity, with a 95% CI that did not cross zero.

Meta-analysis of children’s QoL (Figure 3B) showed no significant effect at post-intervention (SMD = −0.09, 95% CI = −0.26 to 0.09; *z* = 0.99, *p* = .32; *I*^2^ = 42%). There was a low degree of heterogeneity observed (*I^2^* = 42%), which could indicate less variability in measures used and between studies included in the analysis.

The overall effects of the interventions for secondary outcomes and other variables that were reported in individual studies are summarized in Table 5. Effects on parental mental health and parent/family QoL were mixed, while parenting behavior and self-efficacy, child behavior and adjustment, pruritus, and children’s itching-scratching cognitions were significantly improved at post-intervention. There was a general trend of improvement in most outcome variables across the studies.

**Table 5.** Summary of reported effects for secondary outcomes and other outcomes at post-intervention and summary of intervention effects.

Study	Secondary outcomes—parent and family				Other outcomes			
	Parenting skills/efficacy	Effect (Y/N)	Mental health and QoL	Effect (Y/N)	Parent	Effect (Y/N)	Child	Effect (Y/N)
Brunner et al. (2023)	–	–	Parent QoL	N	Fear of topical corticosteroids	Y	–	–
Heapy et al. (2022)	–	–	Parenting stress	Y	–	–	–	–
			Stress	Y				
			Depression	N				
			Anxiety	Y				
			Parent QoL	N				
Kupfer et al. (2010)	Parenting behavior	Y	–	–	–	–	Coping^a^	Y
							Itching-scratching cognitions^a^	Y
Liang et al. (2018)	–	–	-	-	Knowledge of emollients	Y	–	–
Morawska et al. (2016)	SE—AD mgt	Y	Parent HRQoL	Y	Competence with AD mgt	N	Behavior difficulties	Y
	SE—behavior mgt	Y	Family HRQoL	Y				
Morawska et al. (2017)	SE—AD mgt	Y	Parent HRQoL	N	–	–	Behavior difficulties	Y
	SE—behavior mgt	N	Family HRQoL	N				
Pustišek et al. (2016)	–	–	Stress	Y	–	–	Pruritus	Y
			Anxiety—state	Y			Sleep disturbance	Y
			Anxiety—trait	N				
			Parent QoL	Y				
Santer et al. (2022)	–	–	–	–	Enablement	Y	Eczema control	N
					Barriers to treatment use	N		
Staab et al. (2002)	–	–	Parent QoL	N	Treatment use & condition mgt	Y	–	–
			Parent coping	N				
Staab et al (2006)	–	–	Parent QoL	Y	–	–	Itching-scratching cognitions^a^	Y
Weber et al. (2008)	–	–	Family QoL	N	–	–	Pruritus	Y
Xie et al. (2020)	–	–	-	-			Anxiety^a^	Y
							Emotion regulation^a^	Y
							Self-esteem^a^	N
							Relationship with parents^a^	N

*Note*. *HRQoL* = health-related quality of life; *N* = no—no improvement at post-intervention relative to comparison group (where applicable); *QoL* = quality of life; *SE* = self-efficacy; *Y* = yes—improvement at post-intervention relative to comparison group (where applicable).

a Child self-report.

## Discussion

This systematic review sought to summarize the research evidence for psychosocial interventions in the pediatric dermatology context. Only 12 articles reporting on 10 studies were found, all reporting on interventions for children with AD and their families. This highlights an important gap in the existing research literature on the efficacy of psychosocial interventions for families of children with other dermatological conditions.

### Disease/symptom severity

The available evidence suggests that psychosocial interventions and interventions incorporating psychosocial intervention components may help to improve clinical outcomes for children with AD, with results of meta-analysis indicating a small- to medium-sized overall effect on disease/symptom severity. Despite a wide range of effect sizes for individual studies (*d* = .12–.83), seven out of eight effects on disease severity did favor the intervention groups. These results align with meta-analyses reporting small- to moderate-sized effects of psychosocial interventions on health outcomes for children with chronic pain (Fisher et al., 2014) and obesity (Janicke et al., 2014).

While most studies reported small effect sizes (*d* < .50), the mean differences between intervention and control groups for the only two studies reporting large effects (Pustišek et al., 2016; Xie et al., 2020) exceeded the minimum clinically important difference on the disease severity measure (SCORAD), indicating group-level effects that are clinically important. Interestingly, these two interventions were among the briefest (2 h; Pustišek et al., 2016) and lengthiest (18 h; Xie et al., 2020) of the interventions included in this review. This suggests that while longer interventions can be effective, brief interventions may be comparably so. Given that intervention length tends to correlate with the cost of delivery, this is an important consideration for health service delivery.

Although Putisek et al.’s brief 2-h parent-focused structured education program (2016) included information/discussion on psychosocial topics and relaxation strategies, it predominantly focused on AD education. In contrast, Xie et al.’s lengthier intervention (6 × 3-h group sessions, delivered in parallel to parents and children) was a stand-alone mind-body-spirit intervention (2020) without a specific AD education component. This suggests that comparable results may be achieved with brief or lengthy interventions, with or without child inclusion in intervention delivery, and provides some latitude for future research and clinical delivery to adopt the format that is most likely to result in uptake. Nevertheless, given the heterogeneity in interventions and the small number of studies, more research is needed to understand effective intervention components and efficient delivery modalities.

### Child QoL and secondary outcomes

In alignment with previous systematic reviews of psychosocial interventions for children with chronic health conditions, effects on child QoL and secondary outcomes were mixed. While significant effects were fairly consistent within some categories of secondary outcomes (e.g., parenting skills/self-efficacy, other child outcomes), most outcomes were assessed in only one study. Others improved in some studies but not others (e.g., parent/family QoL, parent mental health), and analyses were often underpowered. Future research requires sufficiently large samples to produce definitive results for these outcomes, potentially engaging multiple sites to ensure sufficient population access. Use of the Harmonising Outcome Measures for Eczema (Leshem et al., 2023) Core Outcome Set and Core Outcome Instruments to guide future study design would ensure that all clinical trials for AD include the minimum set of agreed-upon measures for clinical signs, patient-reported symptoms, long-term control, and QoL, to ease comparisons between studies, enable meta-analyses, and improve the quality, consistency, and generalizability of research in this area moving forward.

Most studies were either conducted in Europe or Australia with predominantly Caucasian/White samples. Few studies reported on robust indicators of socioeconomic status, none reported on gender identity or disability status, and no studies undertook subgroup analyses based on sociodemographic characteristics. Whether sociodemographic factors influenced intervention outcomes within or between studies, and whether results are generalizable across different socioeconomic and cultural contexts, is therefore unknown. The absence of studies conducted in Africa or North America is notable, and underscores the need to prioritize research with children and families across diverse geographic, cultural, and sociodemographic contexts to support inclusive, equity-focused research that is generalizable and can be used to improve care for families most in need of support. This is especially important for conditions such as AD where complex biological, environmental, and social factors influence disease onset and trajectory, and context-sensitive intervention development and implementation are important (Croce et al., 2021; Thyssen et al., 2020). Improved understanding of factors influencing intervention development, engagement and effectiveness in different populations and subgroups will support more nuanced evaluations of intervention efficacy and, ultimately, enable health care providers to identify interventions that are effective and appropriate to their own specific patient groups and clinical practice contexts.

### Limitations

This systematic review was limited to studies published in English, and otherwise eligible articles in other languages may have been missed. Despite an exhaustive search, no studies with families of children with dermatological conditions other than AD were found. This makes it impossible to draw any conclusions about the efficacy of psychosocial interventions for children with dermatological conditions more broadly, and research is needed to develop and evaluate psychosocial interventions for other conditions (e.g., psoriasis, vitiligo) where psychosocial factors are a contributor to or a consequence of disease severity and/or treatment difficulties.

Although adherence was not a pre-specified outcome, we used a broad search strategy (no outcome term) to ensure we captured all potentially relevant studies. Effects on competence with AD management, treatment use, and barriers to treatment use were reported as “other” outcomes (Table 5). Future systematic reviews could focus on adherence and/or other secondary outcomes from this review as primary outcomes to generate more reliable estimates of effect. This may be important since improvements in treatment use and more proximal variables (e.g., parent self-efficacy, child behavior) are potentially important mediators of intervention effects on disease/symptom severity.

Most interventions combined condition/treatment education with the psychosocial intervention elements, and it is impossible to disentangle the effects of these different intervention components. Factorial RCT designs, which test each component separately and in combination, or sequential intervention delivery, with education delivered prior to the psychosocial intervention (or vice versa), could help to determine which intervention effects result from each of the individual and combined intervention components. Within the education and psychosocial intervention components, micro-trials (focused on testing one specific intervention process) could further elucidate mechanisms of change (Leijten et al., 2015). The studies also used a wide range of endpoints for post-intervention data collection, meaning that some children were assessed very quickly following intervention commencement (e.g., after 1 week) whereas others had lengthy intervention and/or follow-up periods (e.g., 6–12 months). Thus, the timing of assessment of effects is not consistent across studies and could be expected to affect results, and future studies should plan for longer-term follow-up to assess for maintenance effects.

Finally, four studies were judged as having high risk of bias, predominantly due to lack of blinding of clinicians assessing disease severity. This, combined with issues of imprecision and heterogeneity, downgraded quality of evidence for the disease/symptom severity outcome to “low”. Overall, studies rated as having high risk of bias were less likely to report significant intervention effects for primary and secondary outcomes compared to studies with low risk of bias, and results should be interpreted with caution. Future studies must employ a rigorous study design, including appropriate assessor blinding, to eliminate this foreseeable and preventable risk of bias.

## Conclusion

Evidence suggests that psychosocial interventions may help to improve disease/symptom severity for children with AD. Effects on parenting behavior, parent self-efficacy, child behavior/adjustment and pruritus were also reasonably consistent compared to effects on parent/family QoL and parent mental health, which were inconclusive. A lack of studies for children with other dermatological conditions makes it impossible to draw any conclusions about the efficacy of psychosocial interventions for children with dermatological conditions more broadly. The results of this systematic review offer a mandate to clinical researchers to develop and test psychosocial interventions for children and families struggling with skin disease to improve outcomes that are important to children and families.

## Supplementary Material

jsaf066_Supplementary_Data
